# Systematic review of stigma reducing interventions for African/Black diasporic women

**DOI:** 10.7448/IAS.18.1.19835

**Published:** 2015-04-08

**Authors:** Mona Loutfy, Wangari Tharao, Carmen Logie, Muna A Aden, Lori A Chambers, Wei Wu, Marym Abdelmaseh, Liviana Calzavara

**Affiliations:** 1 Women’s College Research Institute, Women’s College Hospital, Toronto, ON, Canada; 2 Department of Medicine, University of Toronto, Toronto, ON, Canada; 3 Institute of Health Policy, Management and Evaluation, University of Toronto, Toronto, ON, Canada; 4 Research Department, Women’s Health in Women’s Hands, Toronto, ON, Canada; 5 Dalla Lana School of Public Health, University of Toronto, Toronto, ON, Canada; 6 Factor-Inwentash Faculty of Social Work, University of Toronto, Toronto, ON, Canada; 7 School of Social Work, McMaster University, Hamilton, ON, Canada

**Keywords:** HIV, women, African diaspora, stigma, intersectionality, interventions

## Abstract

**Introduction:**

Literature indicates that racism, sexism, homophobia and HIV-related stigma have adverse impacts on health, well-being, and quality of life among HIV-positive women of African descent (African/Black diaspora). However, limited evidence exists on the effectiveness of interventions aimed at reducing stigma tailored for these women. This study systematically reviewed randomized controlled trials (RCTs), non-randomized observational and quasi-experimental studies evaluating the effectiveness of interventions aimed at reducing stigma experienced by this population.

**Methods:**

The Cochrane methodology was used to develop a search strategy in consultation with a librarian scientist. Databases searched included the Cochrane Library, Ovid EMBASE, PsycInfo, and 10 others. Two reviewers independently assessed the studies for potential relevance and conducted the Cochrane grading of RCTs to assess risk of bias and the Newcastle–Ottawa scale to assess the quality of non-randomized studies. Eligible papers were selected if they employed an intervention design with African/Black diasporic women living with HIV as the target population and had a primary outcome of stigma reduction.

**Results:**

Of the five studies that met all of the eligibility criteria, four demonstrated the effectiveness of interventions in reducing HIV-related stigma. Only two of the five studies were designed specifically for HIV-positive African/Black diasporic women. Limitations included the absence of interventions addressing other forms of stigma and discrimination (e.g. gender discrimination, racism, heterosexism).

**Conclusions:**

Our findings suggest that there are limited interventions designed to address multiple forms of stigma, including gender and racial discrimination, experienced by HIV-positive African/Black diasporic women.

## Introduction

Both in the global South and the global North, the HIV epidemic has disproportionately impacted people of African descent. Disproportionate rates of HIV in women of African descent have been reported in Western countries including Canada, the United States and the United Kingdom [1,2]. For the purpose of the introduction, we use Canada as an example of a Western country where a significant proportion of the population is of African descent, making it the third largest racialized group in the country [3]. In reported positive HIV adult test cases from 1998 to 2008, African/Black diasporic women comprised less than 3% of Canadian women yet represented 18.8% of total reported cases [4]. Over one-third, 35.2%, of all AIDS cases among African/Black diasporic populations between 1979 and 2008 were among females, over three times higher than the overall proportion of females in all AIDS cases (9.9%) [5].

The demographic profile in Canada signifies that a high proportion of HIV-positive women were born outside of Canada, particularly in Africa and the Caribbean [3]. Current evidence indicates that immigrant women from countries where HIV is endemic are disproportionately affected by the epidemic. Endemic countries are denoted by the Public Health Agency of Canada as countries where HIV prevalence in the adult population is 1% or greater and where at least one of the following criteria are met: heterosexual contact is attributed to ≥50% of HIV-positive cases; a male to female ratio of 2:1 or less, or HIV prevalence of ≥2% among women receiving prenatal care [3]. Between 1998 and 2009, 51.8% of Canadian HIV-positive cases were categorized under the HIV-endemic exposure category of which the vast majority (95.8%) identified as being of African ancestry [4,6]. However, immigration alone cannot explain the high prevalence of HIV or AIDS in this population since testing services provided to immigrants and refugees have been able to identify HIV-seropositive cases prior to entry into Canada. Notably, a study conducted in the United Kingdom determining HIV acquisition in immigrant populations estimated that 31% of the HIV-positive Black Africans adults sampled acquired HIV while living in the United Kingdom [7]. These findings suggest that an increasing proportion of African/Black diasporic immigrants and refugees acquire HIV post-migration. Furthermore, staggering health inequities – poverty and economic dependency, housing instability and homelessness, underemployment, unemployment, or precarious employment, social isolation and exclusion – underscore the necessity for understanding – and addressing – the social determinants driving the disproportionate impact of HIV among African/Black diasporic women. Particularly, social exclusion stemming from stigma and discrimination has been identified as a key determinant to health inequities in African/Black diasporic women with HIV [8].

A preponderance of research has documented negative associations between singular forms of marginalization – HIV-related stigma, racism, sexism – and the health and well-being of people with HIV [9]. For HIV-positive individuals, stigma and discrimination have been associated with psychosocial, physical and mental health factors including social isolation, anxiety, depression, suicidal ideation and harmful effects on physical and mental health [10–20]. Substantial literature has documented the links between racism and deleterious health outcomes [21–24]. Sexism has also been associated with poor mental health outcomes and can be a contributor to physical and mental health inequities [25–29]. Among African/Black diasporic women, marginalization, stigma and discrimination related to gender, race, class, HIV status or other social identities have been identified as increasing vulnerability to HIV infection, reducing access to care, exacerbating mental health outcomes and diminishing quality of life [8,9,30–33]. While most stigma research has focused on individual factors implicated in producing stigma such as the social rejection, stereotyping, labelling and discrimination enacted by individuals towards those with a socially devalued attribute, there has also been an increasing focus on intersectionality, the ways in which social identities (e.g. ethnoracial identity, gender, sexuality) converge to produce unique forms of marginalization [34–36].

The term intersectional stigma was coined to describe the co-occurrence of stigma based on multiple identities of women living with HIV, including ethnoracial identity, gender and gender identity, national identity, sexual orientation and HIV serostatus [37]. Intersectional stigma is concerned with the multiple, simultaneous and dynamic interchanges among categories of social difference as it interlinks with power and privilege, and systemic oppression and its operation at the micro (interpersonal, intrapersonal), mesa (community, social networks, social norms and practices) and macro (cultural institutions, societal structures) levels [10,38,39]. The intersectionality discourse was popularized by Kimberle Crenshaw in her critical race scholarship theorizing identity, social differentiation and modes of oppression [35]. More recently, HIV and other health research has moved towards intersectionality as a framework to understand health inequities as informed by multiple categories of difference that include but is not exclusive to HIV status, gender, race, class, sexuality and national identity [37,38,40,41].

As recent HIV research recommends interventions that address stigma/discrimination at the micro, mesa and macro levels, there is limited evidence of interventions that address intersecting forms of marginalization [42,43]. Nevertheless, the literature on interventions designed to address stigma/discrimination related to gender, race or HIV-seropositivity indicate that these interventions engage in similar strategies as they focus on: (1) intrapersonal (e.g. counselling, peer support, consciousness raising), (2) interpersonal (e.g. care and support, educational interventions), (3) community (e.g. social media), (4) institutional (e.g. health care policy) and (5) structural/social justice based approaches (e.g. legislative intervention, social action) [22,44–52] to combat stigma.

While evidence syntheses have been conducted evaluating the effectiveness of interventions designed to reduce HIV-related stigma [53,54], there is limited research evaluating stigma reducing interventions specifically designed for African/Black diasporic women with HIV. A review conducted by Brown *et al*. [45] evaluated interventions designed to reduce HIV-related stigma. A second review conducted by Mahajan *et al*. [46] examined the research evidence on HIV and stigma, with HIV-related stigma reducing interventions as one of the areas of interest. While both studies included interventions where stigma reduction was a component of the intervention, the interventions identified only targeted HIV-related stigma; they did not consider other forms of marginalization that may intersect with HIV-related stigma and discrimination such as gender, race, class or sexual identity. Current evidence indicates that although there exist a number of interventions aimed at reducing stigma either as a primary or secondary outcome, the majority of these interventions do not employ an intersectional lens of stigma.

To identify interventions designed for African/Black diasporic women with HIV and to examine their effectiveness at reducing stigma, we reviewed randomized controlled trials (RCTs), non-randomized quasi-experimental and observational studies that reported on the efficacy of stigma reducing interventions compared to treatment as usual in reducing the multiple forms of stigma and discrimination experienced by HIV-positive African/Black diasporic women. For the purposes of this review, we define African/Black diaspora as people of African ancestry who are living outside of the African continent [55] with a specific focus on women living in high-income countries. This definition includes recent emigrants of African nativity, and descendants of African people dispersed through historic movements (e.g. Atlantic slave trade, post-colonial migration, etc.).

## Methods

### Objectives

For our systematic review of stigma reducing interventions for African/Black diasporic women, we identified intervention research that targeted any level (e.g. individual, community, institutional) or form (e.g. HIV-related, gender, racial) of stigma and discrimination experienced by this population. As applicable, we also evaluated the effectiveness of stigma reducing interventions at addressing well-being and self-efficacy among African/Black diasporic women living with HIV.

### Protocol and registration

This project was not prospectively registered. A protocol was developed during the planning process (Grant# CIHR No. 97106 U of T Fund No. 487453 sub-grant 11).

### Literature search strategy

This systematic review adheres to guidelines outlined by the Cochrane Collaboration [56]. The PRISMA flow diagram and checklist were used [57,58]. In consultation with a librarian, a search strategy (available in the Supplementary Appendix) was developed to locate all relevant observational studies and RCTs. Our search strategy did not limit inclusion based on geographic jurisdiction.

In June 2013 (current end date of electronic search), we conducted a search of 13 electronic databases that covered national and international literature in medical/health sciences, psychology, and social sciences. The search strategy was applied to Ovid MEDLINE (1946–current) and Ovid EMBASE (1980–current), then adapted for AgeLine Database (1978 – current), ASSIA: Applied Social Sciences Index and Abstract database (1987 – current), CINAHL (1980 – current), Clinicaltrials.gov (1999 – current), the Cochrane Library, a collection of six databases including the Cochrane Database of Systematic Reviews (CDSR), Cochrane Central Register of Controlled Trials (CENTRAL), Cochrane Methodology Register (CMR), Database of Abstracts of Reviews of Effects (DARE), Health Technology Assessment (HTA) Database, and NHS Economic Evaluation Database (NHS EED) (1898 – current), Dissertation Abstract International (1637 – Current), PsycINFO (1806 – present), Social Services Abstracts database (1979 – current), Social Science Abstracts (1972 – current), Sociological Abstracts (1952 – current), Social Sciences Citation Index (1900 – current). Keywords searched included: (1) HIV/AIDS keywords (e.g. “Human immunodeficiency virus,” “HIV transmission”); (2) stigma/discrimination keywords (e.g. “stigma,” “discrimination,” “homophobia,” “racism,” “sexism,” “stereotyping,” “prejudice”; (3) intervention search terms (e.g. “intervention,” “health education,” “counselling,” “psychoeducational” (see Supplementary Fig. 1 for search strategy used for MEDLINE (OVID)). We also undertook a hand search of 19 journals (Supplementary Appendix) from 1995 to August 2013 to identify articles missed by our search, and conducted a citation search of the reference list of included studies. As only published manuscripts were included, authors were contacted by email to ascertain if a complete manuscript or publication was available. If any additional information or clarification were required, authors were also contacted.

### Study selection

Two reviewers (MAA, MA) divided and screened all titles from the literature search for obvious exclusions. The remaining abstracts were independently assessed by two reviewers (MAA, MA), with disagreements resolved by a third reviewer (LAC). We included published intervention studies if: (1) study population included HIV-positive women of African ancestry (e.g. African, Afro-Caribbean, African–American, Black Canadian, Black European, African Diaspora); (2) the intervention aimed to reduce stigma and discrimination; (3) the study provided quantitative outcome data for: (a) stigma and discrimination (e.g. perceived, internalized, enacted, symbolic, courtesy, layered stigma or intersectional stigma; systemic racism, sexism, classism, violence, social exclusion, health inequity), and (b) well-being (e.g. mental health, physical health, HIV disease management/disease progression, treatment, access to care, health behaviours, etc.), or (4) self-efficacy (e.g. coping, mastery). Only RCTs and observational study designs were included.

### Data extraction

Three reviewers (MAA, WW and LAC) designed and independently piloted a data extraction form. The following items were extracted: (1) study information (study jurisdiction, study design, study setting, enrolment period, total participants enrolled, African/Black diasporic participants enrolled, total analyzed, follow-up duration and total follow-up); (2) participant information (age, sexual orientation, level of education, and relationship status); (3) intervention information (type of intervention, duration of intervention, and mode of delivery); (4) outcomes and measures used. Two investigators (MAA, WW) independently extracted data from relevant research. The definition of stigma reduction intervention is any intervention for which the reduction of stigma or discrimination was considered an outcome of the intervention. Our definition of stigma included HIV-related stigma as well as other forms of stigmatization or discrimination identified (e.g. sexism, racism, heterosexism, sexual stigma). The datasets were compared for each study, and a third party settled disagreements (LAC). Our review did not have any jurisdiction restrictions; though we were only able to extract data from papers published in English.

### Risk of bias assessment

The risk of bias of each eligible study was assessed by two investigators (MAA, WW) using the eight-item Newcastle–Ottawa Scale for non-random and observational studies [59] and the Cochrane Risk of Bias tool [60] for RCTs (Supplementary Appendix). The datasets were compared, and a third party settled disagreements (LAC).

### Data analysis

An a priori decision was made to conduct a meta-analysis; however, due to the heterogeneity of the study findings, including type and measurement of health outcomes, it prevented us from pooling the results using meta-analysis. Instead, we reported on study findings and conducted a descriptive analysis based on reported outcomes.

## Results and discussion

Our study selection process is described in Supplementary Fig. 2 using an adapted version of the PRISMA flow diagram [58]. After removal of duplicate references, 10,931 records from our search of electronic databases were identified. After screening of title and abstracts, 16 records were deemed potentially relevant and retrieved as full-length articles for detailed review. We excluded 11 records for not meeting review criteria: two studies were excluded for not reporting on a stigma/discrimination reduction intervention, five due to study design and four for not reporting stigma/discrimination outcomes. After excluding studies based on intervention, design and outcomes of interest, five studies were identified that met all inclusion criteria [61–65].

The details of the study characteristics of the included studies are reported in Supplementary Table 1. Of the five studies that met criteria for inclusion, three reported findings from RCTs [61,62,64], and two studies were prospective cohort studies [63,65]. All of the studies included in this review were conducted in the United States.

The review sample totalled 238 participants of which 188 participants (79%) identified as African–American women with HIV. Sample size ranged from 11 to 109 per study. Although all of the included studies focused on HIV-positive populations and considered cultural differences such as gender and ethnoracial identity in their analysis, only two of the five studies designed their interventions specifically for African–American women [64,65]. The remaining three interventions were tested on women [61,62] or youth [63] and included African/Black diasporic women in their sample.

All five of the included studies reported on the effectiveness of interventions where stigma reduction was an expected outcome. Abel *et al*. [62] and Abel [61] evaluated the efficacy of an Emotional Writing Disclosure (EWD) intervention for women with HIV; EWD is a cognitive intervention designed to foster the reframing of traumatic events such as the traumatic impact of stigma/discrimination in order to reduce the negative thoughts and emotions associated with such events. Hosek *et al*. [63] evaluated Project ACCEPT (Adolescents Coping, Connecting, Empowering and Protecting Together), a behavioural intervention guided by social cognitive theory that was designed to enhance HIV-knowledge, coping, social support and psychological skills building of youth recently diagnosed with HIV. Miles *et al*. [64] evaluated the effectiveness of a maternal HIV self-care symptoms management intervention designed to improve HIV-related knowledge, reduce emotional distress and to promote self-care and care seeking strategies, among low-income African–American mothers with HIV. Rao *et al*. [65] evaluated the African–American adaptation of the HIV stigma toolkit, a stigma reduction intervention originally developed by the International Centre for Research on Women (ICRW). The African–American adaptation of the intervention incorporated participatory educational exercises designed to counter misinformation around HIV, raise awareness of HIV-related stigma and develop skills to addressing stigmatizing situations within one’s daily life.

### Assessment of risk of bias and data from individual studies

The results of the risk of bias assessments are reported in Supplementary Table 2 for the included RCTs and Supplementary Table 3 for observational studies. Overall, the three RCTs had a moderate risk of bias. Of the two prospective cohort studies Hosek *et al*. [63] had a low risk of bias; Rao *et al*. [65] had an unclear risk of bias as a large number of participants were lost at follow-up.

### Summary of findings

We conducted a descriptive synthesis of findings based on stigma reduction outcomes and reported on the effectiveness of the interventions for reducing stigma and discrimination. We also summarized findings on physical and mental well-being as reported in each study. The resulting data from each included study are presented in Supplementary Table 4.

All five included studies reported stigma outcomes. Four studies measured perceived HIV stigma (i.e. awareness of social devaluation, social rejection, diminished social identity and limited social opportunity attributed to stigma) [61–64]. One study measured internalized stigma (i.e. holding negative views of oneself) [65]. Three studies measured outcomes of well-being including depressive symptomology [62–64], health-related quality of life [62,64], health distress [64], number of infections [64] and mood and affective state [64]. One study measured self-efficacy outcomes including disclosure self-efficacy, sexual discussion self-efficacy and coping [63]. Other outcomes that were measured were cognitive reorganization [61,62], HIV symptomology [62], adherence behaviours [62], side effects from antiretroviral drugs [62] and HIV/AIDS knowledge [63].

Findings from four of the included studies indicated that the specified interventions demonstrated promise in reducing stigma. Both of the studies with an exclusively African/Black diasporic female sample reported reductions in stigma post-intervention. Rao *et al*.’s [65] evaluation of the African–American adaptation of the HIV stigma toolkit found that the stigma scores of participants decreased significantly at both day 2 and day 8 post-intervention when compared to baseline scores. Participants of the maternal HIV-self-care symptoms management intervention evaluated by Miles *et al*. [64] reported reductions in perceived stigma six-month post-intervention.

The two studies with a mixed ethnoracial female sample also demonstrated reductions in stigma post-intervention. Findings from Abel *et al*. [62] and Abel [61] indicated differences in stigma scores following initial treatment between the experimental group and control group, with decreased stigma scores reported for participants in the EWD intervention immediately after intervention, 4 weeks [62], and 12 weeks post-intervention [61].

The one study with a gender and ethnoracially diverse sample demonstrated mixed results in regards to stigma reduction. The Project ACCEPT intervention evaluated by Hosek *et al*. [63] reported significant gender differences in regards to perceived stigma. While measures of perceived stigma had improved overtime for male participants, research findings indicated increased personalized stigma and negative self-image for women at three-month follow-up [63].

Findings on physical and mental well-being post-intervention were mixed. Abel *et al*. [62] reported limited findings related to changes in psychological and physical health post-intervention. In regards to health outcomes, Miles *et al*. [64] reported reductions in depression/dejection one month post-intervention, and reduction in tension/anxiety, and higher physical functioning six months after the intervention was delivered. Similar to their results on stigma outcomes, Hosek *et al*. [63] noted significant gender differences in regards to depressive symptomology, self-efficacy, and social support. The study reported improvements in self-efficacy for sexual discussions, and proactive coping for the female participants post-intervention. While depressive symptoms did not demonstrate a significant improvement for women, overall female participants demonstrated lower levels of depression at baseline compared to their male counterparts.

We performed a comprehensive systematic review of the literature to evaluate the efficacy of current HIV-interventions in reducing stigma and discrimination experienced by African/Black diasporic women with HIV. We identified five studies that met our inclusion criteria, of which four studies demonstrated that their interventions had a positive effect on reducing HIV-related stigma in women living with HIV. These findings indicate that stigma reducing interventions can be of benefit for African/Black diasporic women with HIV. These findings could be useful for practitioners wishing to identify interventions that are effective in addressing HIV-related stigma in this population and the subsequent health benefits of such interventions.

Of these studies, two exclusively sampled African/Black diasporic women with HIV and developed interventions that were culturally appropriate for this population. Rao *et al*. [65] adapted a globally used HIV stigma reduction tool so that it was culturally appropriate for African–American women living with HIV as it considered the social context in which these women lived; Miles *et al*. [64] developed an HIV self-care intervention specifically for maternal caregivers with HIV. As part of their adaptation, study authors conducted qualitative interviews with their particular populations to obtain information that would facilitate cultural appropriateness of their intervention specific to African/Black diasporic women with HIV. Cultural considerations made to these interventions involved the integration of cultural strengths (e.g. African proverbs, pictures and videos of African–American women within teaching material), multi-modality of intervention (e.g., use of video, role playing [65]; oral and written delivery [64]), peer facilitation and supports, attentiveness to disclosure, and addressing participant burden (e.g. flexibility of delivery, home delivery of the intervention). Both interventions also considered the socio-cultural contexts of stigma such as the role of family within African–American women’s lives and its potential for mitigating or fuelling stigma.

The included studies identified interventions that showed promise in reducing the effects of stigma on African/Black diasporic women with HIV; however, only two studies were designed specifically for this population. While Abel *et al*. [62] and Abel [61] conducted interventions that were specific to women, ethnoracial differences were not reported in findings. Though the EWD intervention was informed by a model of health promotion specific to people with HIV, there was limited indication that it was specially adapted for African/Black diasporic women with HIV. The Project ACCEPT intervention, designed for youth, indicated significant gender differences in stigma and health outcomes [63]. Although, the study sample was ethnoracially diverse, ethnoracial differences were not reported. The study findings from Hosek *et al*. [63] revealed that additional evidence is required to determine the feasibility of using this intervention with African/Black diasporic women.

Furthermore, only one of these interventions was specifically designed to address both internal and perceived experiences of stigma. Rao *et al*.’s [65] intervention integrated educational activities and coping mechanisms to navigate stigmatizing situations and to mitigate its emotional effects. While the other studies identified stigma reduction as an outcome of the intervention, it was not the primary focus of the intervention. For Hosek *et al*. [63], the aim of the intervention was to support coping mechanisms after receiving an HIV-diagnosis. The intervention reported in Miles *et al*. [64] addressed self-care symptom management including the potential deleterious impacts of stigma on health and self-care. The EWD intervention aimed to help participants reframe traumatic experiences, such as stigma; however, this intervention did not incorporate strategies to combat enacted stigma in one’s daily life [60,61]. Overall, these interventions focused on helping individuals cope with stigma or its effects on one’s health, well-being and self-care, but there was limited evidence to suggest that these interventions, other than Rao et al. [65] integrated strategies that would facilitate navigation of stigma.

This review also shows limited evidence of the long-term effectiveness of stigma reducing interventions for African/Black diasporic women with HIV. The follow-up duration ranged from four weeks to six months, with only two studies having a follow-up period of three months or longer [61,64]. While these findings demonstrated that stigma reducing interventions could have an immediate or short-term impact on the reduction of stigma, there was insufficient evidence of the long-term effect of these interventions in mitigating stigma.

Additionally, while all of these studies reported HIV-related stigma outcomes using similar measures, there is a lack of data demonstrating the effectiveness of stigma reducing interventions addressing other forms of stigma experienced by HIV-positive African/Black diasporic women including sexism and racism. These interventions were also focused on addressing stigma at the interpersonal or intrapersonal level; there is little evidence of interventions addressing stigma and discrimination at community, institutional or structural levels, which has been identified in the literature as essential to combating stigma [43]. Moreover, all of the included studies were derived from the United States; given the potential differences in other geographic contexts such as incidence and prevalence of HIV and immigration histories of African/Black diasporic populations, more evidence is required to determine the applicability of these interventions to other contexts. While our original interest was to address the needs of African/Black diasporic women living with HIV in Canada, our review highlights the state of the evidence on stigma reducing interventions for this population and results may be relevant for African diasporic populations in other regions.

Several limitations in our review merit discussion. First, although our study did not exclude based on language, in our analysis we were unable to include studies published in languages other than English. Our search strategy did not include an electronic database search for grey literature, though we did conduct manual searches that could capture non-peer reviewed or unpublished literature. Our study definitions of stigma/discrimination and health derived from Westernized concepts and may not have captured non-Westernized conceptualizations of these terms. Lastly, we were unable to pool the data for meta-analysis because of the significant heterogeneity in many study features.

## Conclusions

In summary, our systematic review contributes to the emerging body of literature on stigma reducing interventions for people with HIV [45,53,54]. Notably, it has identified interventions that are promising in reducing stigma experienced by African/Black diasporic women with HIV. Our review is salient given the prevalence of HIV in African/Black diasporic women, coupled with the deleterious impact of stigma on their well-being. While this review identified emerging stigma reducing interventions for this population, our findings argue that there is a lack of stigma reducing interventions designed to address the intersectional stigma and discrimination experienced by African/Black diasporic women with HIV. Only five studies were found that addressed stigma among African/Black diasporic women with HIV, and all of these were conducted in the U.S. This highlights the need to pilot studies with African/Black women with HIV in other high-income geographical contexts, including Canada, the U.K. and Western Europe as well as middle-income regions with African/Black diasporic women (e.g. Central and South America). More intervention research is needed to develop and evaluate the effectiveness of anti-stigma interventions designed for intersectional stigma and discrimination at the individual, community and structural levels.

## Supplementary Material

Systematic review of stigma reducing interventions for African/Black diasporic women

Systematic review of stigma reducing interventions for African/Black diasporic women
