# Supplementary material for: Systematic review of stigma reducing interventions for African/Black diasporic women
Source: J Int AIDS Soc. 2015 Apr 8;18(1):19835. doi: 10.7448/IAS.18.1.19835 (PMC4393416; doi:10.7448/IAS.18.1.19835)
Supplement: Systematic review of stigma reducing interventions for African/Black diasporic women [file JIAS-18-19835-s002.pdf]

Appendix B Search strategies used for review

The following search strategies were conducted for identifying both published and unpublished literature: 1) electronic database searches; 2) hand searches of reference lists from related reviews and articles.

Each search strategy included key search terms for HIV and AIDS, stigma and discrimination, and interventions. There were no data, jurisdiction, or language restrictions used in our search strategy.

**HIV/AIDS**	HIV Infections OR "HIV" or "human immunodeficiency syndrome" OR "Acquired Immune Deficiency Syndrome" OR "Acquired Immunodeficiency Syndrome"
**Stigma/Discrimination**	Stigma OR HIV stigma, or racism OR sexism OR homophobia OR intersectional stigma OR intersectionality OR stereotyping OR shame OR discrimination OR prejudice OR fear OR stigma reduction OR marginalization OR exclusion OR isolation
**Intervention**	Intervention OR experiment OR randomized control trial OR quasi experimental OR Observational OR Cohort OR Cross-sectional OR Case-Control OR Pre-test OR Post-test OR study OR program OR project OR workshop OR evidence based OR health education OR counselling OR therapy OR support OR coping OR mass media OR communication OR multimedia OR policy OR training OR advocacy OR legal OR health care OR psycho-educational

Search Strategies

Electronic database search

First, we conducted a search of 13 electronic databases that covered national and international literature (with no language restrictions) in medical/health sciences, psychology, and social sciences. The search strategy was applied to Ovid MEDLINE (1946-current) and Ovid EMBASE (1980-current), then adapted for AgeLine Database (1978 – current), ASSIA: Applied Social Sciences Index and Abstract database (1987 – current), CINAHL (1980 – current),

Clinicaltrials.gov (1999 – current), the Cochrane Library*(1898 – current), Dissertation Abstract International (1637 - Current), PsycINFO (1806 – present), Social Services Abstracts database (1979 – current), Social Science Abstracts (1972 – current), Sociological Abstracts (1952 – current), Social Sciences Citation Index (1900 – current).

Manual Searches

Thirdly, we conducted a number of manual searches for literature: 1) we conducted hand searches of reference lists from related reviews and articles; and 2) we conduct hand searchers of 16 journals with a particular focus on our topic area (i.e., publications on health, public, health, HIV, or African diasporic populations). Manual searches allowed us to yield literature that may not be as accessible from electronic sources such as unpublished literature. From this search, we yielded 21 additional articles that were not included in our electronic database search.

Database	#
AgeLine	254
ASSIA	1,211
CINAHL	2,395
Clinical Trials.gov	32
Cochrane Library	3,433
Dissertations	905
EMBASE	5,693
MEDLINE	5,656
PsycINFO	3,015
Social Science Abstracts	463
Social Service Abstracts	1,197
Social Science Citation Index	4,272
Sociological Abstracts	1,510
Manual Searches	10
**Total References**	**30,046**
**Duplicates**	**(19,115)**
**Total Included in Title and Abstract Review**	**10,931**

* The Cochrane library is a collection of six databases: Cochrane Database of Systematic Reviews (CDSR), Cochrane Central Register of Controlled Trials (CENTRAL), Cochrane Methodology Register (CMR), Database of Abstracts of Reviews of Effects (DARE), Health Technology Assessment (HTA) Database, and NHS Economic Evaluation Database (NHS EED).

Search Strategy for Electronic Databases

MEDLINE (Ovid)

> Database: Ovid MEDLINE(R) Daily Update <May 29, 2013>, Ovid MEDLINE(R)

> In-Process & Other Non-Indexed Citations and Ovid

> MEDLINE(R) <1946 to Present>

> Search Strategy:

> --

> 1 exp HIV Infections/ (221903)

> 2 (hiv or human immunodeficiency syndrome).tw. (218713)

> 3 (Acquired Immune Deficiency Syndrome or Acquired Immunodeficiency Syndrome or AIDS).tw. (121816)

> 4 or/1-3 (320157)

> 5 social discrimination/ or homophobia/ or racism/ or sexism/ or social stigma/ or stereotyping/ or prejudice/

> (29329)

> 6 (Stigma or racism or sexism or homophobia or intersectionality or stereotyp$ or shame or discrimination or

> prejudice or fear or stigma reduction or marginali#ation or exclusion or isolation).tw. (372567)

> 7 5 or 6 (390336)

> 8 exp counseling/ (31638)

> 9 (intervention or program or programme or project or workshop or evidence based or health education or counselling

> or counseling or therapy or support or coping or mass media or

> communication or multimedia or policy or training or advocacy or legal or health care or healthcare or psycho-educational or psychoeducational).tw. (2894021)

> 10 exp mass media/ or communications media/ (38597)

> 11 health education/ or psychoeducation/ (50839)

> 12 or/8-11 (2958498)

> 13 4 and 7 and 12 (5656)

EMBASE

>

> Database: Embase <1980 to 2013 Week 21> Search Strategy:

> --

> 1 exp Human immunodeficiency virus infection/ (290875)

> 2 human immunodeficiency virus infected patient/ (16194)

> 3 (hiv or human immunodeficiency syndrome or Acquired Immune Deficiency Syndrome or Acquired Immunodeficiency

> Syndrome or AIDS).tw. (330850)

> 4 or/1-3 (396404)

> 5 stigma/ (2904)

> 6 social stigma/ (974)

> 7 social discrimination/ or racism/ or sexism/ (3784)

> 8 (Stigma or racism or sexism or homophobia or intersectionality or stereotyp$ or shame or discrimination or

> prejudice or fear or stigma reduction or marginali#ation or exclusion or isolation).tw. (420963)

> 9 or/5-8 (424229)

> 10 4 and 9 (11963)

> 11 exp counseling/ (96430)

> 12 social support/ (52508)

> 13 health education/ or psychoeducation/ (75342)

> 14 exp mass communication/ (338889)

> 15 (intervention or program or programme or project or workshop or evidence based or health education or counselling

> or counseling or therapy or support or coping or mass media or

> communication or multimedia or policy or training or advocacy or legal or health care or healthcare or psycho-educational or psychoeducational).tw. (3563554)

> 16 or/11-15 (3887755)

> 17 10 and 16 (5693)

>

> ***************************

AgeLine

	Search ID#	Search Terms	Search Options	Actions
	S4	(intervention or program or programme or project or workshop or evidence based or health education or counselling or counseling or therapy or support or coping or mass media or communication or multimedia or policy or training or advocacy or legal or health care or healthcare or psycho-educational or psychoeducationa) AND (S1 AND S2 AND S3)	Search modes - Boolean/Phrase	View Results (254) View Details Edit
	S3	intervention or program or programme or project or workshop or evidence based or health education or counselling or counseling or therapy or support or coping or mass media or communication or multimedia or policy or training or advocacy or legal or health care or healthcare or psycho-educational or psychoeducationa	Search modes - Boolean/Phrase	View Results (80,083) View Details Edit
	S2	Stigma or racism or sexism or homophobia or intersectionality or stereotyp* or shame or discrimination or prejudice or fear or stigma reduction or marginalization or marginalisation or exclusion or isolation	Search modes - Boolean/Phrase	View Results (8,804) View Details Edit
	S1	(hiv or hiv infection* or human immunodeficiency syndrome) OR (aids or acquired immune deficiency syndrome or acquired immunodeficiency syndrome)	Search modes - Boolean/Phrase	View Results (3,291) View Details Edit

ASSIA

Set	Search	Databases	Results
S12	(hiv or aids or human immunodeficiency syndrome or acquired immune deficiency syndrome) AND (all(Stigma) or all(racism) or all(sexism) or all(homophobia) or all(intersectionality) or all(stereotyp*) or all(shame) or all(discrimination) or all(prejudice) or all(fear) or all(stigma reduction) or all(marginalisation) or all(marginalization) or all(exclusion) or all(isolation)) AND (all(intervention) or all(program) or all(programme) or all(project) or all(workshop) or all(evidence based) or all(health education) or all(counselling) or all(counseling) or all(therapy) or all(support) or all(coping) or all(mass media) or all(communication) or all(multimedia) or all(policy) or all(training) or all(advocacy) or all(legal) or all(health care) or all(healthcare) or all(psycho-educational) or all(psychoeducational))	Applied Social Sciences Index and Abstracts (ASSIA)	1211°
S11	all(intervention) or all(program) or all(programme) or all(project) or all(workshop) or all(evidence based) or all(health education) or all(counselling) or all(counseling) or all(therapy) or all(support) or all(coping) or all(mass media) or all(communication) or all(multimedia) or all(policy) or all(training) or all(advocacy) or all(legal) or all(health care) or all(healthcare) or all(psycho-educational) or all(psychoeducational)	Applied Social Sciences Index and Abstracts (ASSIA)	304802*
S10	all(Stigma) or all(racism) or all(sexism) or all(homophobia) or all(intersectionality) or all(stereotyp*) or all(shame) or all(discrimination) or all(prejudice) or all(fear) or all(stigma reduction) or all(marginalisation) or all(marginalization) or all(exclusion) or all(isolation)	Applied Social Sciences Index and Abstracts (ASSIA)	29870*
S9	hiv or aids or human immunodeficiency syndrome or acquired immune deficiency syndrome	Applied Social Sciences Index and Abstracts (ASSIA)	20590*

Set	Search	Databases	Results
S8	(SU.EXACT.EXPLODE("Christian counselling" OR "Cognitive behavioural counselling" OR "Cognitive group counselling" OR "Computer assisted counselling" OR "Computer assisted vocational counselling" OR "Counselling" OR "Crosscultural counselling" OR "Educational guidance" OR "Genetic counselling" OR "Group counselling" OR "Humanistic counselling" OR "Long term counselling" OR "Mandatory counselling" OR "Marriage guidance" OR "Multicultural counselling" OR "Outplacement services" OR "Pastoral counselling" OR "Peer group counselling" OR "Philosophical counselling" OR "Premarital counselling" OR "Pretest counselling" OR "Psychodynamic counselling" OR "Re-evaluation counselling" OR "Rehabilitation counselling" OR "Religious counselling" OR "Selfcounselling" OR "Vocational counselling") OR SU.EXACT("Social support") OR (SU.EXACT("Psychoeducational group therapy") OR SU.EXACT("Psychoeducational treatment") OR SU.EXACT("Psychoeducational support groups")) OR (SU.EXACT("Television") OR SU.EXACT("Communication") OR SU.EXACT.EXPLODE("Academic journals" OR "Advertisements" OR "Animation" OR "Broadcasting" OR "Cable television" OR "Children's magazines" OR "Closed circuit television" OR "Comics" OR "Commercial television" OR "Consumer magazines" OR "Dialogue journals" OR "Digital television" OR "Documentary films" OR "Educational films" OR "Electronic newspapers" OR "Erotic films" OR "Farming magazines" OR "Fashion magazines" OR "Feminist periodicals" OR "Films" OR "Gangster films" OR "Horror films" OR "Interactive television" OR "Literary journals" OR "Live television" OR "Local press" OR "Local radio" OR "Local television" OR "Mass media" OR "Medical journals" OR "Men's magazines" OR "Newspapers" OR "Periodicals" OR "Personal advertisements" OR "Popular magazines" OR "Posters" OR "Press" OR "Problem pages" OR "Radio" OR "Religious broadcasting" OR "Satellite television" OR "Silent films" OR "Student newspapers" OR "Suspense films" OR "Tabloid newspapers" OR "Television" OR "War films" OR "Western films" OR "Women's magazines" OR "Young people's magazines" OR "Young women's magazines"))) OR (intervention or program or programme or project or workshop or evidence based or health education or counselling or counseling or therapy or support or coping or mass media or communication or multimedia or policy or training or advocacy or legal or health care or healthcare or psycho-educational or psychoeducational)	Applied Social Sciences Index and Abstracts (ASSIA)	307843*

Set	Search	Databases	Results
S7	intervention or program or programme or project or workshop or evidence based or health education or counselling or counseling or therapy or support or coping or mass media or communication or multimedia or policy or training or advocacy or legal or health care or healthcare or psycho-educational or psychoeducational	Applied Social Sciences Index and Abstracts (ASSIA)	304802*

Set	Search	Databases	Results
S6	SU.EXACT.EXPLODE("Christian counselling" OR "Cognitive behavioural counselling" OR "Cognitive group counselling" OR "Computer assisted counselling" OR "Computer assisted vocational counselling" OR "Counselling" OR "Crosscultural counselling" OR "Educational guidance" OR "Genetic counselling" OR "Group counselling" OR "Humanistic counselling" OR "Long term counselling" OR "Mandatory counselling" OR "Marriage guidance" OR "Multicultural counselling" OR "Outplacement services" OR "Pastoral counselling" OR "Peer group counselling" OR "Philosophical counselling" OR "Premarital counselling" OR "Pretest counselling" OR "Psychodynamic counselling" OR "Re-evaluation counselling" OR "Rehabilitation counselling" OR "Religious counselling" OR "Selfcounselling" OR "Vocational counselling") OR SU.EXACT("Social support") OR (SU.EXACT("Psychoeducational group therapy") OR SU.EXACT("Psychoeducational treatment") OR SU.EXACT("Psychoeducational support groups")) OR (SU.EXACT("Television") OR SU.EXACT("Communication") OR SU.EXACT.EXPLODE("Academic journals" OR "Advertisements" OR "Animation" OR "Broadcasting" OR "Cable television" OR "Children's magazines" OR "Closed circuit television" OR "Comics" OR "Commercial television" OR "Consumer magazines" OR "Dialogue journals" OR "Digital television" OR "Documentary films" OR "Educational films" OR "Electronic newspapers" OR "Erotic films" OR "Farming magazines" OR "Fashion magazines" OR "Feminist periodicals" OR "Films" OR "Gangster films" OR "Horror films" OR "Interactive television" OR "Literary journals" OR "Live television" OR "Local press" OR "Local radio" OR "Local television" OR "Mass media" OR "Medical journals" OR "Men's magazines" OR "Newspapers" OR "Periodicals" OR "Personal advertisements" OR "Popular magazines" OR "Posters" OR "Press" OR "Problem pages" OR "Radio" OR "Religious broadcasting" OR "Satellite television" OR "Silent films" OR "Student newspapers" OR "Suspense films" OR "Tabloid newspapers" OR "Television" OR "War films" OR "Western films" OR "Women's magazines" OR "Young people's magazines" OR "Young women's magazines"))	Applied Social Sciences Index and Abstracts (ASSIA)	17798*

Set	Search	Databases	Results
S5	(SU.EXACT("HIV") OR SU.EXACT("AIDS") OR (human immunodeficiency syndrome OR hiv OR AIDS or acquired immune deficiency syndrome OR acquired immunodeficiency syndrome)) AND (((SU.EXACT("Gender stereotypes") OR SU.EXACT("Cultural stereotypes") OR SU.EXACT("Racial stereotypes")) OR SU.EXACT("Stigmatization") OR SU.EXACT.EXPLODE("Antiracism" OR "Antisemitism" OR "Antisexism" OR "Heterosexism" OR "Homophobia" OR "Institutional racism" OR "Prejudice" OR "Racial hatred" OR "Racial prejudice" OR "Racism" OR "Sexism" OR "Xenophobia")) OR ((SU.EXACT("Double discrimination") OR SU.EXACT("Racial discrimination") OR SU.EXACT("Intergroup discrimination") OR SU.EXACT("Sex discrimination")) OR (Stigma OR racism OR sexism OR homophobia OR intersectionality OR stereotyp* OR shame OR discrimination OR prejudice OR fear OR stigma reduction OR marginalisation OR marginalization OR exclusion OR isolation)))	Applied Social Sciences Index and Abstracts (ASSIA)	1393°
S4	((SU.EXACT("Gender stereotypes") OR SU.EXACT("Cultural stereotypes") OR SU.EXACT("Racial stereotypes")) OR SU.EXACT("Stigmatization") OR SU.EXACT.EXPLODE("Antiracism" OR "Antisemitism" OR "Antisexism" OR "Heterosexism" OR "Homophobia" OR "Institutional racism" OR "Prejudice" OR "Racial hatred" OR "Racial prejudice" OR "Racism" OR "Sexism" OR "Xenophobia")) OR ((SU.EXACT("Double discrimination") OR SU.EXACT("Racial discrimination") OR SU.EXACT("Intergroup discrimination") OR SU.EXACT("Sex discrimination")) OR (Stigma OR racism OR sexism OR homophobia OR intersectionality OR stereotyp* OR shame OR discrimination OR prejudice OR fear OR stigma reduction OR marginalisation OR marginalization OR exclusion OR isolation))	Applied Social Sciences Index and Abstracts (ASSIA)	30240*
S3	(SU.EXACT("Double discrimination") OR SU.EXACT("Racial discrimination") OR SU.EXACT("Intergroup discrimination") OR SU.EXACT("Sex discrimination")) OR (Stigma or racism or sexism or homophobia or intersectionality or stereotyp* or shame or discrimination or prejudice or fear or stigma reduction or marginalisation or marginalization or exclusion or isolation)	Applied Social Sciences Index and Abstracts (ASSIA)	29870*

Set	Search	Databases	Results
S2	(SU.EXACT("Gender stereotypes") OR SU.EXACT("Cultural stereotypes") OR SU.EXACT("Racial stereotypes")) OR SU.EXACT("Stigmatization") OR SU.EXACT.EXPLODE("Antiracism" OR "Antisemitism" OR "Antisexism" OR "Heterosexism" OR "Homophobia" OR "Institutional racism" OR "Prejudice" OR "Racial hatred" OR "Racial prejudice" OR "Racism" OR "Sexism" OR "Xenophobia")	Applied Social Sciences Index and Abstracts (ASSIA)	4859*
S1	SU.EXACT("HIV") OR SU.EXACT("AIDS") OR (human immunodeficiency syndrome or hiv or AIDS or acquired immune deficiency syndrome or acquired immunodeficiency syndrome)	Applied Social Sciences Index and Abstracts (ASSIA)	13641*

CINAHL

Search ID#	Search Terms	Actions
S18	S15 AND S16 AND S17	View Results (2,395)
S17	S7 OR S8 OR S9 OR S10 OR S11 OR S12	View Results (812,779)
S16	S3 OR S14	View Results (68,233)
S15	S4 OR S5 OR S6 OR S13	View Results (49,006)
S14	TI (hiv or human immunodeficiency syndrome or Acquired Immune Deficiency Syndrome or Acquired Immunodeficiency Syndrome or AIDS) OR AB (hiv or human immunodeficiency syndrome or Acquired Immune Deficiency Syndrome or Acquired Immunodeficiency Syndrome or AIDS)	View Results (49,455)
S13	TI (Stigma or racism or sexism or homophobia or intersectionality or stereotyp* or shame or discrimination or prejudice or fear or stigma reduction or marginalization or marginalisation or exclusion or isolation) OR AB (Stigma or racism or sexism or homophobia or intersectionality or stereotyp* or shame or discrimination or prejudice or fear or stigma reduction or marginalization or marginalisation or exclusion or isolation)	View Results (36,578) View Details Edit
S12	TI (intervention or program or programme or project or workshop or evidence based or health education or counselling or counseling or therapy or support or coping or mass media or communication or multimedia or policy or training or advocacy or legal or health care or healthcare or psycho-educational or psychoeducational) OR AB (intervention or program or programme or project or workshop or evidence based or health education or counselling or counseling or therapy or support or coping or mass ...	View Results (594,052) View Details Edit
S11	(MH "Support, Psychosocial+")	View Results (35,046)
S10	(MH "Psychoeducation")	View Results (1,276)

Search ID#	Search Terms	Actions
S9	(MH "Health Education")	View Results (12,895)
S8	(MH "Communications Media+")	View Results (263,219)
S7	(MH "Counseling+")	View Results (15,502)
S6	(MH "Homophobia")	View Results (562)
S5	(MH "Stigma") OR (MH "Stereotyping") OR (MH "Prejudice")	View Results (9,876)
S4	(MH "Discrimination") OR (MH "Racism") OR (MH "Sexism+")	View Results (10,392)
S3	S1 OR S2	View Results (68,233)
S2	hiv or human immunodeficiency syndrome or Acquired Immune Deficiency Syndrome or Acquired Immunodeficiency Syndrome or AIDS	View Results (68,233)
S1	(MH "HIV Infections+")	View Results (44,620)

Clinical Trials.gov

Search of: (HIV OR AIDS) AND (stigma OR prejudice OR sexism OR racism…phobia) | Interventional Studies - List Results - ClinicalTrials.gov 2013-06-02 12:30 PM

No Query Suggestions

Recognized Terms and Synonyms:

homophobia: 1 studies
anti gay bias
anti homosexual bias

prejudice: 6 studies

racism: 5 studies
race and ethnic discrimination
racial bias
racial discrimination
racial prejudices

sexism: 3 studies
biased gender
gender bias
sex bias

stigma: 100 studies

aids: 6322 studies
acquired immun-deficiency synd
acquired immune deficiency
acquired immuno deficiency syndrome
aids disorders
aids virus
autoimmune deficiency syndrome
diseases due to immunodeficiency virus
hiv
htlv-iii
human immunodeficiency virus
human t cell lymphotropic virus type iii
human t lymphotropic virus type iii

human t-cell leukemia virus type iii
immune deficiency syndrome acquired aids
lymphadenopathy associated virus

hiv: 5936 studies
acquired immun-deficiency synd
acquired immune deficiency
acquired immuno deficiency syndrome
aids disorders
aids virus
autoimmune deficiency syndrome
diseases due to immunodeficiency virus
htlv-iii
human immunodeficiency virus
human t cell lymphotropic virus type iii
human t lymphotropic virus type iii
human t-cell leukemia virus type iii
immune deficiency syndrome acquired aids
lymphadenopathy associated virus

32 studies found for: (HIV OR AIDS) AND (stigma OR prejudice OR sexism OR racism OR homophobia) | Interventional Studies

Cochrane Library (Cochrane Database of Systematic Reviews, Cochrane Central Register of Controlled Trials, Cochrane Methodology Register, Database of Abstracts of Reviews of Effects, Health Technology Assessment Database, and NHS Economic Evaluation Database)

Search Name:
Date Run: 02/06/13 16:04:51.723

Description:

ID

Search Hits

#1 MeSH descriptor: [HIV Infections] explode all trees 7096
#2 hiv or human immunodeficiency syndrome or Acquired Immune Deficiency Syndrome or Acquired Immunodeficiency Syndrome or AIDS 18997

#3 #1 or #2 18997

#4 MeSH descriptor: [Sexism] explode all trees 2

#5 MeSH descriptor: [Social Discrimination] this term only 1
#6 MeSH descriptor: [Prejudice] explode all trees 240
#7 MeSH descriptor: [Stereotyping] explode all trees 222
#8 MeSH descriptor: [Social Stigma] explode all trees 10
#9 Stigma or racism or sexism or homophobia or intersectionality or stereotyp* or shame or discrimination or prejudice or fear or stigma reduction or marginalization or marginalisation or exclusion or isolation 27526
#10 #4 or #5 or #6 or #7 or #8 or #9 27526
#11 #3 and #10 3520

#12 MeSH descriptor: [Counseling] explode all trees 2783
#13 MeSH descriptor: [Social Support] explode all trees 2044

#14 MeSH descriptor: [Mass Media] explode all trees 1365

#15 MeSH descriptor: [Communications Media] explode all trees 5981
#16 MeSH descriptor: [Health Education] explode all trees 8788
#17 intervention or program or programme or project or workshop or evidence based or health education or counselling or counseling or therapy or support or coping or mass media or communication or multimedia or policy or training or advocacy or legal or health care or healthcare or psycho-educational or psychoeducational 472502

#18 #12 or #13 or #14 or #15 or #16 or #17 473098
#19 #11 and #18 3433

Dissertations

((all(human immunodeficiency virus OR hiv infection) OR ((all(aids) and (all(hiv) or all(human immunodeficiency virus))) or all(acquired immune deficiency syndrome))) AND (all(Stigma) or all(racism) or all(sexism) or all(homophobia) or all(intersectionality) or all(stereotyp*) or all(shame) or all(discrimination) or all(prejudice) or all(fear) or all(stigma reduction) or all(marginalisation) or all(marginalization) or all(exclusion) or all(isolation))) AND (all(intervention) or all(program) or all(programme) or all(project) or all(workshop) or all(evidence based) or all(health education) or all(counselling) or all(counseling) or all(therapy) or all(support) or all(coping) or all(mass media) or all(communication) or all(multimedia) or all(policy) or all(training) or all(advocacy) or all(legal) or all(health care) or all(healthcare) or all(psycho-educational) or all(psychoeducational))

PsycINFO

Database: PsycINFO <1806 to May Week 4 2013> Search Strategy:

> --

> 1 exp hiv/ (29514)

> 2 (hiv or human immunodeficiency syndrome or Acquired Immune Deficiency Syndrome or Acquired Immunodeficiency

> Syndrome or AIDS).tw. (47236)

> 3 1 or 2 (47345)

> 4 stigma/ (5242)

> 5 social discrimination/ or disability discrimination/ or "race and ethnic discrimination"/ or sex discrimination/

> (7231)

> 6 racism/ (4961)

> 7 sexism/ (1456)

> 8 prejudice/ or stereotyped attitudes/ (13917)

> 9 (Stigma or racism or sexism or homophobia or intersectionality or stereotyp$ or shame or discrimination or

> prejudice or fear or stigma reduction or marginali#ation or exclusion or isolation).tw. (183783)

> 10 or/4-9 (188879)

> 11 3 and 10 (4880)

> 12 exp counseling/ (63688)

> 13 social support/ (25268)

> 14 psychoeducation/ (2974)

> 15 exp communications media/ (43733)

> 16 (intervention or program or programme or project or workshop or evidence based or health education or counselling

> or counseling or therapy or support or coping or mass media or

> communication or multimedia or policy or training or advocacy or legal or health care or healthcare or psycho-educational or psychoeducational).tw. (1100955)

> 17 or/12-16 (1135070)

> 18 11 and 17 (3015)

>

> ***************************

Social Science Abstracts

Search ID#	Search Terms	Actions
S12	S5 AND S11	View Results (463) View Details Edit
S11	S6 OR S7 OR S8 OR S9 OR S10	View Results (399,526) View Details Edit
S10	intervention or program or programme or project or workshop or evidence based or health education or counselling or counseling or therapy or support or coping or mass media or communication or multimedia or policy or training or advocacy or legal or health care or healthcare or psycho-educational or psychoeducational	View Results (399,236) View Details Edit
S9	DE "Communication" OR DE "Mass media" OR DE "Social media"	View Results (1,365) View Details Edit
S8	DE "Health education"	View Results (187) View Details Edit
S7	(DE "Social support")	View Results (3,211) View Details Edit

S6	DE "Counseling" OR DE "Assertiveness training" OR DE "Counselor & client" OR DE "Counselors" OR DE "Cross-cultural counseling" OR DE "Educational counseling" OR DE "Family counseling" OR DE "Group counseling" OR DE "LGBT hotlines (Counseling)" OR DE "Marriage counseling" OR DE "Mentoring" OR DE "Motivational interviewing" OR DE "Sex counseling" OR DE "Vocational guidance"	View Results (3,282) View Details Edit
S5	S1 AND S4	View Results (683) View Details Edit
S4	S2 OR S3	View Results (50,408) View Details Edit
S3	Stigma or racism or sexism or homophobia or intersectionality or stereotyp* or shame or discrimination or prejudice or fear or stigma reduction or marginalisation or marginalization or exclusion or isolation	View Results (50,408) View Details Edit
S2	(DE "Stigma (Social psychology)") OR (DE "Discrimination" OR DE "Discrimination against people with AIDS" OR DE "Discrimination in banking" OR DE "Discrimination in capital punishment" OR DE "Discrimination in education" OR DE "Discrimination in employment" OR DE "Discrimination in housing" OR DE "Discrimination in insurance" OR DE "Discrimination in justice administration" OR DE "Discrimination in law enforcement" OR DE "Discrimination in medical care" OR DE "Discrimination in mental health ser ...	View Results (3,861) View Details Edit
S1	human immunodeficiency syndrome or hiv or AIDS or acquired immune deficiency syndrome or acquired immunodeficiency syndrome	View Results (7,252) View Details Edit

Social Service Abstracts

((all(human immunodeficiency virus OR hiv infection) OR ((all(aids) and (all(hiv) or all(human immunodeficiency virus))) or all(acquired immune deficiency syndrome))) AND (all(Stigma) or all(racism) or all(sexism) or all(homophobia) or all(intersectionality) or all(stereotyp*) or all(shame) or all(discrimination) or all(prejudice) or all(fear) or all(stigma reduction) or all(marginalisation) or all(marginalization) or all(exclusion) or all(isolation))) AND (all(intervention) or all(program) or all(programme) or all(project) or all(workshop) or all(evidence based) or all(health education) or all(counselling) or all(counseling) or all(therapy) or all(support) or all(coping) or all(mass media) or all(communication) or all(multimedia) or all(policy) or all(training) or all(advocacy) or all(legal) or all(health care) or all(healthcare) or all(psycho-educational) or all(psychoeducational))

Social Science Citation Index

# 5	4,272	#4 AND #3<br><br>Databases=SSCI Timespan=All years
.		
# 4	1,240,367	TS=(intervention or program or programme or project or workshop or evidence based or health education or counselling or counseling or therapy or support or coping or mass media or communication or multimedia or policy or training or advocacy or legal or "health care" or healthcare or psycho-educational or psychoeducational) or TI=(intervention or program or programme or project or workshop or evidence based or health education or counselling or counseling or therapy or support or coping or mass media or communication or multimedia or policy or training or advocacy or legal or "health care" or healthcare or psycho-educational or psychoeducational)<br><br>Databases=SSCI Timespan=All years
.		
# 3	5,912	#2 AND #1<br><br>Databases=SSCI Timespan=All years
.		

# 2	137,157	TS=(stigma or racism or sexism or homophobia or intersectionality or stereotyp* or shame or discrimination or prejudice or fear or marginalization or marginalisation or exclusion or isolation) or TI=(stigma or racism or sexism or homophobia or intersectionality or stereotyp* or shame or discrimination or prejudice or fear or marginalization or marginalisation or exclusion or isolation)<br><br>Databases=SSCI Timespan=All years
.		
# 1	83,168	Topic=(hiv or "human immunodeficiency syndrome or Acquired Immune Deficiency Syndrome" or "Acquired Immunodeficiency Syndrome" or AIDS) OR Title=(hiv or "human immunodeficiency syndrome or Acquired Immune Deficiency Syndrome" or "Acquired Immunodeficiency Syndrome" or AIDS)<br><br>Databases=SSCI Timespan=All years

Sociological Abstracts

((all(human immunodeficiency virus OR hiv infection) OR ((all(aids) and (all(hiv) or all(human immunodeficiency virus))) or all(acquired immune deficiency syndrome))) AND (all(Stigma) or all(racism) or all(sexism) or all(homophobia) or all(intersectionality) or all(stereotyp*) or all(shame) or all(discrimination) or all(prejudice) or all(fear) or all(stigma reduction) or all(marginalisation) or all(marginalization) or all(exclusion) or all(isolation))) AND (all(intervention) or all(program) or all(programme) or all(project) or all(workshop) or all(evidence based) or all(health education) or all(counselling) or all(counseling) or all(therapy) or all(support) or all(coping) or all(mass media) or all(communication) or all(multimedia) or all(policy) or all(training) or all(advocacy) or all(legal) or all(health care) or all(healthcare) or all(psycho-educational) or all(psychoeducational))

Manual Searchers

Relevant Journals for Handsearch

1. American Journal of Health Education (2007-present)
   - HIV AIDS Stigma→Interventions→Women→AA Women
     - No new relevant articles
2. African and Black Diaspora (2008-present)
   - HIV OR AIDS
     - No new relevant articles
3. African Diaspora (2008 to present)
   - HIV OR AIDS + Stigma
     - no new relevant articles
4. African Health Sciences (2001-present)
   - HIV Stigma
     - No new relevant articles
5. African journal for the psychological study of social issues (2004-present)
   - HIV Stigma
     - No new relevant articles
6. African journal of AIDS research (2004-present)
   - HIV Stigma
     - No new relevant articles
7. AIDS (01.01.1988 to present)
   - Stigma AND intervention
     - Effects of cognitive behavioral stress management on HIV-1 RNA, CD4 cell counts and psychosocial parameters of HIV-infected persons. Berger, Simona AIDS. 22(6):767-775, March 30, 2008.
   - Stigma reduc* intervention
     - No new relevant articles
   - Women AND stigma
     - No new relevant articles
   - Women AND intervention
     - No new relevant articles

8. AIDS alert (2008-12.2011)

- HIV Stigma
  - No new relevant articles

9. AIDS and Behavior (1999-present)

- HIV Stigma→Intervention→AA→Women
  - No new relevant articles

10. AIDS care (1996 to present)

- Stigma AND women AND intervention
  - No new relevant articles
- Stigma AND intervention AND HIV
  - Reducing AIDS-related stigma in developing countries: The importance of theory- and evidence-based interventions. Arjan E.R. Bos (Psychology, Health & Medicine (August 2008), 13 (4), pg. 450-460)….good for informing intervention NOT a quantitative study
- Stigma AND intervention AND HIV AND women
  - No new relevant articles

11. AIDS Education and Prevention (2002-present)

- HIV Stigma Reduction
  - No new relevant articles

12. AIDS Patient care and STDs (1996-present)

- Stigma AND intervention AND HIV
  - No new relevant articles

13. AIDS Research and Therapy (2006-present)

- HIV Stigma
  - No new relevant articles

14. British Journal of Psychotherapy

- HIV OR AIDS AND Stigma AND intervention
  - No new relevant articles

15. British Journal of Social Work

- HIV Stigma + Reduction
  - No new relevant articles

16. Canadian Journal of Counseling and Psychotherapy

- HIV Stigma
  - No new relevant articles

17. Counselling and Psychotherapy Research (01.04.2001 to present)

- HIV AND stigma AND intervention
  - No new relevant articles
- HIV
  - No new relevant articles

18. Social Science and Medicine (1995-present)

- HIV Stigma→Interventions→Women→Black
  - No new relevant articles

19. Stigma Research and Action

- HIV Stigma
    - No new relevant articles

Grey Literature Sources[a]

1. Weiss, M. and Ramakrishna, J. (2001). *Interventions: Research on Reducing Stigma*. Retrieved from: http://www.stigmaconference.nih.gov/WeissPaper.htm
2. Engender Health. (2004). *Reducing Stigma and Discrimination Related to HIV/AIDS-Training for Health Care Workers*. Retrieved from: http://www.engenderhealth.org/files/pubs/hiv-aids-stis/reducing_stigma_trainer_english.pdf (training manual--thought parts of it would be interesting for intervention development—more for background than anything)
3. UNAIDS. (2007). *Reducing HIV/AIDS Stigma and Discrimination: a critical part of national AIDS programmes. A resource for national stakeholders in the HIV response*. Retrieved from: http://www.unaids.org/en/media/unaids/contentassets/dataimport/pub/report/2008/jc1521_stigmatisation_en.pdf
4. UNAIDS. (2012). *Guidance Note: Key Programmes to Reduce Stigma and Discrimination and Increase Access to Justice in National HIV Responses*. Retrieved from: http://www.unaids.org/en/media/unaids/contentassets/documents/document/2012/Key_Human_Rights_Programmes_en_May2012.pdf
5. Duvvury, N., Prasad, N., Kishore, N. (2006). *HIV/AIDS Stigma and Violence Reduction Intervention Manual—ICRW*. Retrieved from: www.kit.nl/kit/Stigma-and-Violence-Reduction-Intervention-Manual
6. DFID. (2007). Taking Action Against HIV Stigma and Discrimination, Guidance Document and Supporting Resources. Retrieved from http://www.icrw.org/files/publications/DFID-Taking-Action-Against-HIV-Stigma-and-Discrimination.pdf
7. Armitage, D. (2007). Considering Training as an intervention to reduce HIV stigma. *HIV Nursing*, 11-12. Retrieved from: http://go.galegroup.com.myaccess.library.utoronto.ca/ps/i.do?action=interpret&id=GALE%7CA200343534&v=2.1&u=utoronto_main&it=pdf&p=AONE&workId=PI-1TTO-2007-AUT00-IDSI-9.JPG%7CPI-1TTO-2007-AUT00-IDSI-10.JPG&callistoContentSet=PER&sw=w&authCount=1
8. Kidd, R. and Clay, S. (2003). *Understanding and challenging HIV stigma: Toolkit for Action*. Retrieved from: http://www.icrw.org/publications/understanding-and-challenging-hiv-stigma-toolkit-action
9. International Centre for Research on Women. (2006). *HIV/AIDS Stigma. Finding Solutions to Strengthen HIV/AIDS Programs.* Retrieved from: http://www.icrw.org/files/publications/HIV-AIDS-Stigma-Finding-Solutions-to-Strengthen-HIV-AIDS-Programs.pdf
10. De Bryun, T. (2004). A plan of action for Canada to reduce HIV/AIDS related stigma and discrimination—Canadian HIV/AIDS Legal Network. Retrieved from: http://www.aidslaw.ca/publications/interfaces/downloadFile.php?ref=48

***Systematic Reviews for Ancestry Searches**[b]*

Blashill, A. J., Perry, N., & Safren, S. A. Mental health: A focus on stress, coping, and mental illness as it relates to treatment retention, adherence, and other health outcomes.

Brown, L., Macintyre, K., & Trujillo, L. (2003). Interventions to reduce HIV/AIDS stigma: What have we learned?

Heijnders, M., & Van Der Meij, S. (2006). The Fight Against Stigma: An Overview of Stigma-Reduction Strategies and Interventions. Psychology, health & medicine, 11(3), 353-363. doi: http://dx.doi.org/10.1080/13548500600595327

Kaplan, A. H., Scheyett, A., & Golin, C. E. (2005). HIV and stigma: analysis and research program.

Mahajan, A. P., Sayles, J. N., Patel, V. A., Remien, R. H., Sawires, S. R., Ortiz, D. J., . . . Coates, T. J. (2008). Stigma in the HIV/AIDS epidemic: a review of the literature and recommendations for the way forward.

Nyblade, L., Stangl, A., Weiss, E., & Ashburn, K. (2009). Combating HIV stigma in health care settings: what works?

Pulerwitz, J., Michaelis, A., Weiss, E., Brown, L., & Mahendra, V. (2010). Reducing HIV-related stigma: lessons learned from Horizons research and programs.

Sandelowski, M., Barroso, J., & Voils, C. I. (2009). Gender, Race/Ethnicity, and Social Class in Research Reports on Stigma in HIV-Positive Women. Health Care for Women International, 30(4), 273-288. doi: http://dx.doi.org/10.1080/07399330802694880

Sandelowski, M., Lambe, C., & Barroso, J. (2004). Stigma in HIV-poslitive women. Journal of Nursing Scholarship, 36(2), 122-128. doi: 10.1111/j.1547-5069.2004.04024.x

Sengupta, S., Banks, B., Jonas, D., Miles, M. S., & Smith, G. C. HIV interventions to reduce HIV/AIDS stigma: a systematic review.

Smith, R., Rossetto, K., & Peterson, B. L. (2008). A meta-analysis of disclosure of one's HIV-positive status, stigma and social support.

---

a No included literature yielded from grey literature search.

b No new articles were identified that were not available in the database search
